# Genetics of Hypertension in African Americans and Others of African Descent

**DOI:** 10.3390/ijms20051081

**Published:** 2019-03-02

**Authors:** Mihail Zilbermint, Fady Hannah-Shmouni, Constantine A. Stratakis

**Affiliations:** 1 Section on Endocrinology and Genetics, The Eunice Kennedy Shriver National Institute of Child Health and Human Development, National Institutes of Health, BG 31 RM 2A46, 31 Center Dr, Bethesda, MD 20892, USA; Fady.Hannah-Shmouni@nih.gov (F.H.-S.); stratakc@cc1.nichd.nih.gov (C.A.S.); 2 Division of Endocrinology, Diabetes, and Metabolism, Johns Hopkins University School of Medicine, Baltimore, MD 21287, USA; 3 Johns Hopkins Community Physicians at Suburban Hospital, Bethesda, MD 20814, USA; 4 Johns Hopkins University Carey Business School, Baltimore, MD 21202, USA

**Keywords:** genetics, hypertension, African American, low-renin, *ARMC5*, *SCNN1B*, *GRK4*, *CACNA1D*, endocrine hypertension

## Abstract

Hypertension is the leading cause of cardiovascular disease in the United States, affecting up to one-third of adults. When compared to other ethnic or racial groups in the United States, African Americans and other people of African descent show a higher incidence of hypertension and its related comorbidities; however, the genetics of hypertension in these populations has not been studied adequately. Several genes have been identified to play a role in the genetics of hypertension. They include genes regulating the renin-aldosterone-angiotensin system (RAAS), such as Sodium Channel Epithelial 1 Beta Subunit (*SCNN1B*), Armadillo Repeat Containing 5 (*ARMC5*), G Protein-Coupled Receptor Kinase 4 *(GRK4*), and Calcium Voltage-Gated Channel Subunit Alpha1 D (*CACNA1D*). In this review, we focus on recent genetic findings available in the public domain for potential differences between African Americans and other populations. We also cover some recent and relevant discoveries in the field of low-renin hypertension from our laboratory at the National Institutes of Health. Understanding the different genetics of hypertension among various groups is essential for effective precision-guided medical therapy of high blood pressure.

## 1. Introduction

Hypertension is the leading cause of cardiovascular disease in the United States, affecting 29% of adults, or approximately 75 million people [1]. The economic burden of hypertension in America is enormous [2], often raising concerns about its major impact on health disparity [3,4,5]. When compared to other ethnic or racial groups in the United States, African American and others of African descent show a higher incidence of hypertension and its related comorbidities, including cardiovascular and end-stage renal diseases [2]. Moreover, African Americans and others of African descent may have higher blood pressure beginning in childhood, as well as a higher incidence and prevalence of hypertension across the lifespan [6,7,8,9,10,11]. The age-adjusted prevalence of hypertension in African Americans is ~45%, significantly higher than in other ethnicities, including ~32% among non-Hispanic whites and ~30% among Hispanics [2].

Major predictors of hypertension in African Americans exist. For example, impaired arterial elasticity [12,13,14] has been demonstrated to be more prevalent than in whites [5,15,16,17]. Brown et al. found in a large cohort study of untreated normotensive participants (Multi-Ethnic Study of Atherosclerosis) that subjects who were found to have a suppressed renin phenotype were more likely to be African Americans, and had higher systolic blood pressure [18]. In a recent observational study on normotensive African Americans, aldosterone sensitivity (magnitude of the association between plasma aldosterone concentration and blood pressure) increased with age and was associated with plasma aldosterone concentration and the aldosterone-to-renin ratio, suggesting that mineralocorticoid receptor activity may increase with age, especially in African Americans [19]. Other factors that distinguish hypertension in African Americans from other ethnicities include increased awareness of diagnosis, increased intensity of treatment, poor BP control, and more resistant hypertension [20]. On the other hand, hypertensive diagnostic inertia, defined as a failure to investigate the underlying cause of hypertension, is an ongoing issue in African Americans with cardiovascular disease [21,22]. Moreover, adrenocortical hyperplasia was found to be more common in African Americans and other patients of African descent [23], suggesting the possibility of aldosterone and/or cortisol excess as an important contributor to the pathogenesis of hypertension in this ethnic group.

The regulation of blood pressure is complex. Research that examines the association of the various pathophysiological factors with incident hypertension among African Americans and others of African descent is limited, as detailed in Table 1. Despite heredity contributing 40–50% to the pathogenesis of essential hypertension and genome-wide association studies identifying ~6% of the genetic contribution [24,25], little is known about the genetic diversity of hypertension in African American populations, particularly in relation to the factors listed in Table 1. Some researchers have focused on genes implicated in altered renal sodium handling in the kidneys and volume loading, which are key players in the development of low-renin hypertension in this at-risk population [26,27]. Studies that failed to discover any relationship between African American and hypertension were limited by several factors, including variation in allele frequency, small statistical power, and the possibility of weak ancestral effects [28,29,30]. Large sample size studies could exclude > 95% of the genome as harboring risk loci of > 1.3 due to African or European ancestry, further suggesting the complexity of understanding the underpinning of hypertension across various ethnicities. Ongoing studies are examining genetic susceptibility and environmental factors as determinants of hypertension in African Americans [31].

Diversity may exist within African descent population. Clinical and genetic data of African-Americans and others of African descent should be interpreted and compared with caution. Chor et al. studied blood pressure profiles of 15,103 civil servants in Brazil and found a high prevalence of high blood pressure among browns (38.2%) and blacks (49.3%) [11]. Importantly, they found that hypertensive characteristics of Brazilian brown populations were like that seen in the individuals of African descent.

Genetic sequencing has gained enormous popularity in the scientific field. Affordability and fast throughput are promising to deliver “Genetic Health Risk and Carrier Status” for as little as $99, through direct-to-consumer salivary collection kits [43]. However, the clinical interpretation of an individual’s genome, its utility in clinical practice, and the overall cost has yet to be implemented as a standard of care approach in universal clinical management guidelines. One of the major goals of understanding the genetics of hypertension includes the transfer of genomic knowledge into daily clinical practice [10], for potential gene-targeted medical therapies [5], among other useful utilities.

In this review, we briefly highlight the mechanistic and genetic underpinnings of hypertension in African Americans and other populations of African ancestry. We have focused our discussion on the biologically plausible hypertension candidate gene loci in African Americans [44]

## 2. Hypertension in African Americans: Clinical Differences and Genetics

### 2.1. Renin and Aldosterone

African Americans excrete a sodium load more slowly and less completely than whites [45]. This results in suppression of the renin-aldosterone-angiotensin system (RAAS) due to volume-loading that typically begins in childhood [46,47,48,49]. Ultimately, a low-renin state, which compensates for the relative tendency to retain sodium, ensues [50]. Low-renin hypertension is a frequent cause of hypertension, with a prevalence of 20%–30%, and higher in African Americans [51,52,53]. One study demonstrated lower levels of plasma renin activity and aldosterone in normotensive African Americans across all ages, with BP positively correlating with plasma aldosterone, an effect that increased as plasma renin activity decreased [48]. Thus, a typical biochemical profile in an African American person with hypertension is a low or high plasma aldosterone concentration, a low or suppressed plasma renin activity or direct renin concentration, and suppressed angiotensin I and II [36,50,54,55]. This results in a normal or elevated aldosterone-to-renin ratio, which can be categorized into two broad hypertension phenotypes: low or suppressed renin and elevated aldosterone (primary aldosteronism type, or hyporeninemic hyperaldosteronism) and low renin and low aldosterone (Liddle syndrome type, or hyporeninemic hypoaldosteronism) [56]. The threshold set to diagnose a low renin state is assay specific but generally defined as a plasma renin activity < 0.65 ng/mL/h or a direct renin concentration < 15μU/mL [52].

### 2.2. Regulation of Sodium Reabsorbtion

It has been speculated that several genes implicated in the regulation of sodium reabsorption in the kidneys were likely selected as an adaptation to high temperature environments, particularly in people from Sub-Saharan Africa [20,57]. These include genes regulating RAAS, such as (Sodium Channel Epithelial 1 Beta Subunit (*SCNN1B*) and Neural Precursor Cell Expressed, Developmentally Down-Regulated 4 (*NEDD4*) that alter sodium retention from the kidneys, and possibly armadillo repeat containing 5 (*ARMC5*) that might be responsible for increased aldosterone production from the adrenal cortex (see below).

These genetic factors may have played an important physiological adaptation (“natural selection”) to the low sodium environments and survival of African Americans during their passage from Africa to America on ships [20,57], where they witnessed extreme conditions including severe heat, hyperhidrosis, and fluid loss through sickness. Indeed, this selection process may have contributed to the increased prevalence of hypertension in this population [58,59]. In this section, we cover the most important known genetic contributions to hypertension in African Americans.

### 2.3. ENaC Function

The amiloride-sensitive epithelial sodium channel (ENaC) is in the distal nephron and responsible for regulating the amount of sodium reabsorbed by the kidneys, primarily through the action of aldosterone. ENaC is composed of 3 homologous subunits of similar structure and encoded by separate genes: Sodium Channel Epithelial 1 Alpha Subunit (*SCNN1A*) on 12p13.31, Sodium Channel Epithelial 1 Beta Subunit (*SCNN1B*) on 16p12.2, and Sodium Channel Epithelial 1 Gamma Subunit (*SCNN1G*) on 16p12.2. There are two transmembrane domains (TM1 and TM2) and two short intracellular domains (*C*- and *N*-terminus); the C-termini contain a binding site for Nedd4 (neural precursor cell expressed, developmentally down-regulated 4), a ubiquitin E3 ligase protein encoded by *NEED4* on 15q21.3 and responsible for the internalization and the proteasomal degradation of ENaC [60,61,62].

#### 2.3.1. Liddle’s Syndrome

Constitutive activation variants of *SCNN1B* or *SCNN1G* result in salt-sensitive hypertension known as Liddle’s syndrome, an autosomal dominant form of monogenic hypertension that is characterized by early-onset of low-renin hypertension [63]. Patients with Liddle’s syndrome are resistant to mineralocorticoid antagonist therapy but respond to an ENaC inhibitor, such as amiloride therapy [63,64]. Gain-of-function variants in the genes encoding for ENaC are in the carboxyterminal cytoplasmic tail of the protein, which is involved in down-regulation of channel number or activity [50,65]. This area of the nephron is the final regulator of sodium balance and activating variants in ENaC leads to sodium retention, potassium excretion, low renin/aldosterone (hyporeninemic hypoaldosteronism), and volume overload [20,66].

#### 2.3.2. Hyporeninemic Hypoaldosteronism (Liddle Phenotype)

Hyporeninemic hypoaldosteronism not due to Liddle’s syndrome, also referred to as the Liddle phenotype, is more common in African Americans for multiple reasons, including the interplay of certain genes that lead to ethnic differences in proximal and distal tubular sodium reabsorption [67]. Tu et al. confirmed this association between ENaC overreactivity and hypertension in African Americans by demonstrating increased retention of sodium and water after stimulation with 2 weeks of 9-α fludrocortisone [48]. Moreover, ENaC over-activation could also be due to altered internalization and degradation by *NEDD4* and acquired or inherited causes of aldosterone excess. On the other hand, loss-of-function variants in other segments of ENaC cause pseudohypoaldosteronism, an autosomal recessive condition that is characterized by salt-loss and mineralocorticoid resistance [68].

#### 2.3.3. *SCNN1B* and *NEED4*

Activation of ENaC, either due to structural variants of the channel subunits (e.g.,: *SCNN1B*) or altered activity of regulatory processes (including *NEED4*), could underlie low-renin hypertension in African Americans. One study showed that ENaC channel activity, as assessed by nasal transmucosal electrical potential difference, was greater in African Americans than in whites [69]. In a mixed ancestry population of South Africans, a variant in *SCNN1B* (p.R563Q) was present in 18 people, of whom 17 were hypertensive [50]. In another study, this variant was present in 6% of Africans from urban South Africa that responded to treatment with amiloride therapy [70]. This variant was associated with a resistant form of hyporeninemic hypoaldosteronism hypertension, analogous to Liddle syndrome. Moreover, the p.T594M but not p.G442V (which causes lower aldosterone secretion, suggesting increased ENaC activity) variants in *SCNN1B* may also contribute to hypertension in African Americans [71]. In another study, individuals with variants in *NEDD4* were linked to increased BP and adverse cardiovascular outcomes [20,72,73].

#### 2.3.4. ENaC Function, CYP4A11 and Responsiveness to Amiloride Therapy

Individuals with variants affecting ENaC function and hypertension may respond preferentially to amiloride therapy. Studies from salt-sensitive hypertensive rodent models showed decreased expression of *Cyp4a* and increased ENaC activity responsive to amiloride [74,75]. Human studies on African American patients with resistant hypertension demonstrated homozygosity for the C allele at rs3890011 of Cytochrome P450 Family 4 Subfamily A Member 11 *(CYP4A11*) (1p33), which has been previously associated with blood pressure in various populations [30,76], and a positive response to amiloride therapy [77]. A large high-density admixture scan in 1670 African Americans with hypertension identified this locus as a candidate gene for hypertension [30]. *CYP4A11* encodes a member of the cytochrome P450 superfamily of enzymes, a monooxygenase which catalyze reactions involved in the synthesis of cholesterol, steroids, and other lipids and localized to the endoplasmic reticulum. Several lines of evidence suggest that this gene serves as a modulator of ENaC function [75,77], likely through decreased epoxygenase activity and renal synthesis of epoxyeicosatrienoic acids [20,77]. Collectively, although these variants are more frequent in African Americans, their association with hypertension has been weak and/or inconsistent [50,78,79]. Further studies with a larger population size are required to study their effects on hypertension in the African American populations.

### 2.4. Adrenocortical Hyperplasia, Tumors, and Primary Aldosteronism

#### 2.4.1. *KCNJ5*, *CACNA1D*, *ATP1A1*, *ATP2B3*, and *CTNNB1*

Primary aldosteronism is the most common cause of endocrine hypertension [80]. Autonomous secretion of aldosterone from the adrenal glands suppresses endogenous renin production, which results in an increased volume status. The most frequent cause of primary aldosteronism is bilateral adrenal hyperplasia [81], often referred to as idiopathic adrenal hyperplasia. Recent evidence suggests that these lesions harbor areas of hyperplasia due to cytochrome P450 family 11 subfamily B member 2 (CYP11B2)-expressing cells from an unknown germline variants, and at least 1 CYP11B2-positive aldosterone-producing cell cluster (APCC, that typically develops with aging) or micro-aldosterone-producing adenomas, in part due to calcium or potassium channel variants (Calcium Voltage-Gated Channel Subunit Alpha1 D (*CACNA1D*), 58%; Potassium Voltage-Gated Channel Subfamily J Member 5 (*KCNJ5*), 1%) [82].

Recently, Nanba et al. studied the genetic characteristics of 73 aldosterone-producing adrenocortical adenomas in 79 subjects of African American decent who had primary aldosteronism; 65 subjects had somatic alterations in driver genes. The genetic landscape of these tumors was different than in non-African Americans: alteartions in *CACNA1D* (*n* = 42%), *KCNJ5* (34%), ATPase Na+/K+ Transporting Subunit Alpha 1 (*ATP1A1*) (8%), and ATPase Plasma Membrane Ca2+ Transporting 3 (*ATP2B3*) (4%) represented the spectrum [83]. No variants in *ARMC5* were found in this study. These results suggest that *CACNA1D* could be one of the most frequently mutated aldosterone-driver gene in African Americans, suggesting a possible primary role for calcium channel blockers in the management of these individuals.

Familial or inherited causes of primary aldosteronism are rare and caused by disease-causing germline activating variants in several genes as detailed elsewhere [81].

#### 2.4.2. Bilateral Adrenocortical Hyperplasia

Bilateral adrenocortical hyperplasias are grossly divided into the micronodular and macronodular disease. The micronodular subtypes are usually diagnosed in children and young adults and are either pigmented (primary pigmented nodular adrenocortical disease [PPNAD] as seen in Carney complex) or not pigmented. The macronodular subtypes, which are usually diagnosed in adults over the age of 40, may be sporadic or familial and caused by disease-causing variants in *ARMC5*, Adenomatous Polyposis Coli (*APC*), Multiple Endocrine Neoplasia type 1 (*MEN1*), and Fumarate Hydratase (*FH*) [84,85,86]. African Americans with hypertension and a biochemical phenotype of hyporeninemic hyperaldosteronism are more likely to have bilateral adrenal hyperplasia, with or without nodules [84,85,87,88,89,90,91].

#### 2.4.3. *ARMC5*

The *ARMC5* gene is a putative tumor-suppressor that is located on chromosome 16p11.2 and belongs to the family of armadillo (ARM)-repeat-containing proteins. In humans, *ARMC5* consists of 8 exons and has an unknown function. The *ARMC5* gene has been recently implicated in endogenous hypercortisolemia due to a rare form of adrenocortical hyperplasia, termed primary bilateral macronodular adrenal hyperplasia (PBMAH) [84,92,93]. This condition is characterized by multiple macronodules (>1 cm) in the adrenal cortex and hypercortisolemia; it is also rarely associated with primary aldosteronism [85]. Biallelic inactivating variants in *ARMC5* (germline and somatic) are required for the development of adrenocortical hyperplasia, which is consistent with the two-hit hypothesis of tumorigenesis [84,85,92]. Most variants in *ARMC5* are frameshift and/or nonsense, and lead to loss of function of the gene. Our group has recently shown that *Armc5* knockout mice died during early embryonic development, while a third of heterozygotes developed hypercorticosteronemia at 18 months of age [94]. Several pathways may be involved in *Armc5* haploinsufficiency, including cyclic AMP (protein kinase A, its catalytic subunit Cα) and the Wnt/β-catenin pathways [94].

Our laboratory has recently identified an association between biallelic variants of *ARMC5* in African Americans and primary aldosteronism [85]. We hypothesized that these variants likely act as a selective advantage for people of African descent to excrete water more slowly as a survival mechanism in hot climates through enhanced excretion of aldosterone from the adrenal cortex [95]. Our initial studies showed that 20 unrelated and two related study subjects (39.3%) harbored 12 germline *ARMC5* variants that were predicted to be damaging by in silico analysis. Interestingly, all patients carrying a variant predicted to be damaging were African Americans (Table 2).

In a different study, we investigated a large cohort of African Americans in the Minority Health Genomics and Translational Research Bio-Repository Database (MH-GRID) study. The MH-GRID genomic database comprises a large group of subjects with hypertension, both resistant and severe subtypes. We hypothesized that a direct association between *ARMC5* variants and increased risk of hypertension in African American exists. 1377 subjects (mean age: 48.25 (SD ± 6.06), controls 43.35 (SD ± 7.23), P = 1.17×10-40) and 44 variants within *ARMC5* (3 common, 4 low frequency and 37 rare variants) were considered for analysis. *ARMC5* variant rs116201073 reached nominal significance (P = 0.044) and odds ratio (OR) = 0.7, suggesting a protective effect for this variant. A set of 16 rare variants significantly associated with hypertension was identified and combined with the common variant, associated with hypertension in the single-variant analysis, representing a variant set associated with hypertension (*P* = 0.0121). These results confirmed our previous report of increased germline *ARMC5* variants that may be associated with hypertension. Further genetic and molecular studies are needed to confirm these findings [103].

### 2.5. Other genes

This section highlights several variants in genes that have been associated with hypertension in African Americans, which are also listed in Table 2.

#### 2.5.1. *GRK4*

G protein-coupled receptor kinases (GRKs) participate in the desensitization of G protein-coupled receptors, including D1 receptors, in the proximal renal tubules; variants in *GRK4* (4p16.3) were shown to improperly excrete sodium in rodents and humans with hypertension [104]. A recent report using genetic sequencing of genes implicated in sodium and water retention in African Americans revealed variants associated with amino acid changes were implicated with low renin resistant hypertension [105]. Three variants in *GRK4* (pR65L, p.A142V, and p. A486V) were 94.4% predictive of salt-sensitive hypertension. Additionally, the number of *GRK4* variants was inversely related to salt excretion [105], suggesting that *GRK4* is an important gene in the regulation of the hyporeninemic hypoaldosteronism phenotype in African Americans with hypertension. In this study, other variants that were reported in association with hypertension in the same population included *CYP11B2*, which encodes for aldosterone synthase.

#### 2.5.2. *SCG2*, *PHOX2A* and *PHOX2B*

Wen et al. reported on the association between a regulatory variant in secretogranin II (*SCG2*) in African Americans [96]. They sequenced the entire gene from 180 diverse ethnic group and showed that variant 736 was common among subjects of African descent. Two transactivating factors were identified, including ARIX (*PHOX2A*) and *PHOX2B*, while a positive selection of the protective allele within the human lineage was observed [96]. In another study, a single variant that converts methionine to threonine at amino acid 235 (M235T) of the angiotensinogen gene (*AGT*) was found to be associated with hypertension in Caucasians [106]. Kumar et al. reported an A/G polymorphism at position -217 of the *AGT* gene promoter and found that the frequency of allele A is increased in African Americans [97].

#### 2.5.3. *GPR25* and *SMOC1*

The Family Blood Pressure Program, which included 10,798 participants in 3320 families (3962 African Americans) observed a significant linkage on chromosome 7 (logarithm of odds [LOD] = 3.1) in African Americans people from the GenNet Network [98]. Sherva et al. identified loci contributing to arterial stiffness in a cohort 1251 Black people (HyperGEN study) [13]. Scientists identified two regions which were highly suggestive of linkage on chromosome 1 (LOD = 3.08), and another one on chromosome 14 (LOD = 2.42). Two candidate genes ((G Protein-Coupled Receptor 25 (*GPR25*), SPARC Related Modular Calcium Binding 1 (*SMOC1*)) were in the linked regions [13].

#### 2.5.4. *SLC24A4* and *CACNA1H*

The Howard University Family Study studied a panel of over 800,000 variants from 1,017 African Americans from the Washington, D.C., metropolitan region and found two genes (Solute Carrier Family 24 Member 4 (*SLC24A4*) and Calcium Voltage-Gated Channel Subunit Alpha1 H (*CACNA1H*)) as potential candidates for blood pressure regulation [99].

#### 2.5.5. *CYP11B2*

The *CYP11B2* gene encodes for a cytochrome P450 protein, a monooxygenase which catalyzes the synthesis of cholesterol, steroids, and other lipids in the inner mitochondrial membrane; also known as aldosterone synthase, this enzyme has an 18-hydroxylase activity to synthesize aldosterone and 18-oxocortisol as well as steroid 11 beta-hydroxylase activity and is the rate limiting step in aldosterone production [107].

The largest study to date from 3 African countries examined several variants of genes implicated in low renin-resistant hypertension in Africans with suppressed renin and increased aldosterone. Six candidate genes were sequenced, including *CYP11B2*, *SCNN1B*, *NEDD4L*, *GRK4*, Uromodulin (*UMOD*), and Natriuretic Peptide A (*NPPA*) based on the renin-aldosterone status [105]. Fourteen nonsynonymous variants of *CYP11B2* were found, with 3 previously described and associated with alterations in aldosterone synthase production (R87G, V386A, and G435S) [105]. Further studies are required to ascertain these findings.

Apparent mineralocorticoid excess (AME) refers to a rare autosomal recessive disorder leading to low renin hypertension due to alterations in *HSD11B2* (16q22.1), which encodes for the corticosteroid 11-beta-dehydrogenase. This is a microsomal enzyme complex responsible for the interconversion of cortisol and cortisone in the kidneys, thus preventing the activation of the mineralocorticoid receptor by glucocorticoids. Variants in this gene have been associated with essential hypertension [108,109,110], likely through decreased cortisol inactivation to cortisone which is seen with aging, and biochemically confirmed as elevated urinary tetrahydrocortisol (THF, A-ring-reduced cortisol metabolite) + alloTHF to tetrahydrocortisone (THE, cortisol metabolite) [(THF+alloTHF)/THE] [111]. One study demonstrated an association between microsatellite markers close to the *HSD11B2* gene and hypertension in African Americans that also suffered from end-stage renal disease likely due to hypertension [53,112]. Further studies are required to confirm this association with the increased predisposition of African Americans to low-renin, salt-sensitive hypertension.

#### 2.5.6. Other Genetic Variants

Zhu et al. performed admixture mapping for hypertension loci with genome-scan markers from individuals of African descent and European Americans (Family Blood Pressure Program). They found that chromosome 6q24 and 21q21 may contain genes associated with risk of hypertension in African Americans [27]. Another admixture mapping in African Americans was done in the Dallas Heart Study [100]. Researchers genotyped a panel of 2270 variants in a random sample of 1743 African Americans and found a missense variant in Vanin 1 (*VNN1*) (rs2272996) that was significantly associated with hypertension in African Americans. In the Candidate Gene Association Resource (CARe) consortium, a novel variant (rs7726475) on chromosome 5 (between the SUB1 Homolog, Transcriptional Regulator (*SUB1*) and Natriuretic Peptide Receptor 3 (*NPR3*) genes) and rs7726475 was found to be associated with both systolic and diastolic hypertension [101].

Finally, in the International Consortium for Blood Pressure Genome-Wide Association (19,775 subjects of African ancestry) [102], scientists identified a large number of variants associated with either systolic or diastolic hypertension in Africans, including: rs13082711 (*SLC4A7*) (SBP/DBP); rs419076 (*MECOM*), (SBP); rs13107325 (*SLC39A8*) (SBP/DBP); rs13139571 (*GUCY1A3-GUCY1B3*) (SBP/DBP); rs1173771 (*NPR3-C5orf23*) (SBP); rs11953630 (*EBF1*) (SBP/DBP); rs805303 (*BAT2-BAT5*) (DBP); rs7129220 (*ADM*) (SBP/DBP); rs633185 (*FLJ32810/TMEM133*) (SBP); rs2521501 (*FURIN-FES*) (SBP/DBP); rs17608766 (*GOSR2*) (SBP/DBP); rs1327235 (*JAG1*) (SBP/DBP); rs6015450 (*GNAS-EDN3*) (SBP/DBP); rs17367504 (*MTHFR-NPPB*) (SBP/DBP); rs3774372 (*ULK4*) (SBP/DBP); rs1458038 (*FGF5*) (SBP/DBP); rs1813353 (*CACNB2*) (SBP/DBP); rs11191548 (*CYP17A1-NT5C2*) (SBP/DBP); rs381815 (*PLEKHA7*) (SBP/DBP); rs3184504 (*SH2B3*) (SBP/DBP); rs1378942 (*CYP1A1-ULK3*) (SBP): rs12940887 (*ZNF652*) (SBP/DBP).

## 3. Genetic Counselling

To date, the identification of genetic variants that predispose to hypertension in African Americans has not enabled genetic diagnosis and early identification of patients and their at-risk family members. Thus, genetic testing is not currently routine in clinical practice. Indeed, with the exception of *ARMC5* and *CACNA1D* (as outlined above), the other genes discussed in this review have no current clinical implications for the management of hypertension in African Americans. When a clinician encounters a patient with a pathogenic and damaging *ARMC5* variant, screening for Cushing syndrome and primary aldosteronism is encouraged. From our experience, *ARMC5*-related adrenal pathology does not clinically present in early childhood. *ARMC5*-related endocrine hypertension diseases typically develop in adulthood as either subclinical Cushing syndrome, with or without primary aldosteronism, or overt Cushing syndrome. Carriers may not show signs of these conditions until later in adulthood, typically over 40 years of age. Genetic testing and counselling of family members should be considered as the conditions associated with *ARMC5* follow an autosomal dominant inheritance pattern with decreased penetrance.

## 4. Conclusions

Hypertension in African Americans is the leading cause of cardiovascular disease in this population. The complex interactions between genetic and environmental determinants are yet to be identified. Several genes implicated in RAAS activation have been studied in African American populations and have revealed a surprising number of novel variants and pathways possibly implicated in the pathogenesis of hypertension. Among them, variants in the *ARMC5* gene appear to be a rare but inherited cause of primary aldosteronism and consequently low-renin hypertension in African Americans. Further studies are needed to determine the significance of the genes discussed in this review and respective pathways, which will guide personalized precision therapy for hypertension.

## Figures and Tables

**Table 1:** Examples of pathophysiological mechanisms of hypertension in individuals of African descent.

Mechanism
Psychosocial factors [32]
Endothelial dysfunction [33,34]
Kidney injury and function [35]
Renin-angiotensin system activation [36]
Insulin resistance [37]
Impaired baroreflex [38]
Oxidative stress [39]
Genetic factors [20]
Adrenomedullary/cortical hormones [23]
Blood volume [40]
Salt retention [20,41]
Socioeconomic determinants [42]

**Table 2:** Genes associated with pulse pressure, systolic, diastolic blood pressure and hypertension. Adapted by permission from Springer Nature: [Springer Nature] [Journal of Cardiovascular Translational Research] [Hall, J.L.; Duprez, D.A.; Barac, A.; Rich, S.S. A review of genetics, arterial stiffness, and blood pressure in African Americans, 5, 302-308.e), [Springer Science+Business media, LLC] (2012).

Study	Race-ethnicity	Traits	Discovery
Primary aldosteronism NIH study [85]	*ARMC5* variants in AA with PA (*n* = 22)/*ARMC5* variants ESPS6500 Sample (*n* = 2203)	Primary aldosteronism and hypertension	*ARMC5* maps to 16p11.2. rs35461188 (Benign), rs200655247 (Damaging), rs142376949 (Benign),
University of Michigan study and NIH [83]	Black patients from USA, *n* = 79 (73 patients had aldosterone-producing adenoma)	Primary aldosteronism and hypertension	73 adrenocortical tumors from 79 PA patients expressed *CACNA1D* (42%), *KCNJ5* (34%), *ATP1A1* (*n* = 8%), *ATP2B3* (4%).
University of California San Diego/Kaiser/VA/Loyola [96]	AA (n = 383), Combined cohort Kaiser/VA (n = 527), Nigerian cohort Loyola	Systolic Blood Pressure, hypertension	SCG2 (two transactivating factors were identified, ARIX (PHOX2A) and PHOX2B
The State University of NY Health Science Center [97]	AA (n = 342), Caucasians (n = 263)	Systolic Blood pressure	AGT (-217A variant)
Family Blood Pressure Program [98]	AA (*n* = 3962)/Whites (*n* = 3667)/Hispanics (*n* = 1612)/Asian (*n* = 1557) U.S.	Pulse pressure	Chromosome 7 at 75 cM, LOD 3.1 in AA, chromosome 19 at 0 cM LOD 3.1 in combined sample whites and AA, and a region on chromosome 18 at 71 cM LOD score of 3.2 in whites, AA, and Hispanics.
HyperGEN [13]	AA (*n* = 1251) U.S.	Pulse Pressure	Chromosome 1, 215 cM, LOD 3.08, Chromosome 14, 85 cM, LOD 2.42
Howard University Family Study [99]	AA (*n* = 1017) U.S. A 2^nd^ cohort of 980 West Africans	Systolic BP	rs5743185, rs16877320, rs11160059, rs17365948, rs12279202, rs3751664. *SLC24A4* (a sodium/potassium/calcium exchanger) and *CACNA1H* (a voltage-dependent calcium channel),
Family Blood Pressure Program and Nigerian cohort [27]	AA (*n* = 737) (cases) European Americans (*n* = 573) (controls)	Hypertension	5 markers on chromosome 6q (near region 6q24); 2 markers on chromosome 21 (near region 21q21) may contain genes influencing risk of hypertension in AA
Dallas Heart Study [100]	AA (*n* = 1743), White (*n* = 1000), Mexican American (*n* = 581)	Hypertension	rs2272996
Candidate Gene Association Resource Consortum [101]	AA (*n* = 6303), replication Cohorts: Women’s Health Initiative, Maywood, GENOA, Howard University Family Study, native Nigerian African Sample (*n* = 11,882)	Systolic and diastolic blood pressure	rs7726475 between genes *SUB1* and *NPR3*
International Consortium for Blood Pressure Association Studies [102]	AA (*n* = 19,775), Europeans (*n* = 200,000), East Asians (*n* = 29,719), South Asians	Systolic and Diastolic blood pressure, hypertension	rs13082711 (*SLC4A7*) (SBP/DBP); rs419076 (*MECOM*), (SBP); rs13107325 (*SLC39A8*) (SBP/DBP); rs13139571 (*GUCY1A3*-*GUCY1B3*) (SBP/DBP); rs1173771 (*NPR3*-C5orf23) (SBP); rs11953630 (*EBF1*) (SBP/DBP); rs805303 (*BAT2-BAT5*) (DBP); rs7129220 (*ADM*) (SBP/DBP); rs633185 (*FLJ32810/TMEM133*) (SBP); rs2521501 (*FURIN-FES*) (SBP/DBP); rs17608766 (*GOSR2*) (SBP/DBP); rs1327235 (*JAG1*) (SBP/DBP); rs6015450 (*GNAS-EDN3*) (SBP/DBP); rs17367504 (*MTHFR-NPPB*) (SBP/DBP); rs3774372 (*ULK4*) (SBP/DBP); rs1458038 (*FGF5*) (SBP/DBP); rs1813353 (*CACNB2*(3′) (SBP/DBP); rs11191548 (*CYP17A1-NT5C2*) (SBP/DBP); rs381815 (*PLEKHA7*) (SBP/DBP); rs3184504 (SH2B3) (SBP/DBP); rs1378942 (*CYP1A1-ULK3*) (SBP): rs12940887 (*ZNF652*) (SBP/DBP)

## Abbreviations

AA	African Americans
PP	pulse pressure
PA	Primary aldosteronism
SBP	Systolic blood pressure
DBP	Diastolic blood pressure
